# Perception of seismic design by architects in Algeria

**DOI:** 10.4102/jamba.v12i1.864

**Published:** 2020-09-23

**Authors:** Bachir Benfarhat, Djillali Benouar, Stéphane Cartier

**Affiliations:** 1 Department of Civil Engineering, Faculty of Civil Engineering and Architecture, Amar Telidji University of Laghouat, Laghouat, Algeria; 2 Faculty of Civil Engineering, University of Science and Technology Houari Boumediene, Alger, Algeria; 3 Pacte Laboratory, Institute of Urbanism and Alpine Geography, University of Grenoble, Grenoble, France

**Keywords:** Seismic risk, seismic design, seismic code, seismic training, Algerian Paraseismic Regulation (RPA) seismic code

## Abstract

The purpose of this article is to analyse the results of a survey towards the architects and engineers of construction in order to estimate the perception of the anti-seismic protection amongst these professionals. It focuses more on the architects who design buildings and, in many cases, monitor their implementation. The main result is the observation of inadequacies and gaps in seismic culture amongst the professionals, especially architects. These difficulties require the reinforcement of earthquake-resistant training. This effort to upgrade skills is as important as other aspects of the preventive management of the seismic risk in Algeria.

## Introduction

Anti-seismic codes are imposed to reinforce the safety of structures with the application of calculation rules and construction provisions. However, building codes cannot absolutely ensure this safety. Indeed, it has been shown that architectural design is as important as the enforcement of seismic codes. The behaviour of a structure under a shake is practically determined by the geometry, inputting the distribution of masses and rigid elements, and the type of structures (Zacek 1996; Zacek & Balandier 2003).

Seismic design is a joint responsibility of architect and engineer. It is based on the reasoned choice of forms of the building. The adoption of appropriate construction provisions and rigorous control of the implementation on site is also needed.

However, everywhere, there is a gap between progress in knowledge of the ductility of structures and the effectiveness of seismic codes for the project managers (Cartier & Vallette 2016).

Designers, architects and engineers are the first professions concerned by the respect of the principles of earthquake-resistant architecture and the application of earthquake-resistant codes.

In seismic engineering, a design error cannot be caught by calculations, as sophisticated as it may be, and the consequences are often catastrophic (Betbeder-Matibet 2003).

In Algeria, the seismic codes organise the design of buildings, but this is not enough. Each element of the protection is a link in the security chain. This management chain is only as strong as its weakest link (Benouar 2000, 2001).

The Algerian paraseismic rules (RPA) promote vigilance, particularly in the northern part of Algeria where designers must enforce a level of safety to buildings in order to cope with the seismic hazard (CGS 2003).

Nevertheless, seismic design can stimulate architecture without condemning it to simple forms, or opposing the architects’ bold forms. An architect trained in seismic engineering can assert this competence with the project owner (Zacek 2003).

In order to understand better the difficulties of designers with seismic protection, we surveyed this professional audience. This survey attempts to identify their representation of seismic hazard and opportunities for technological adaptation. It is also a way to understand if the training corresponds to their needs and skills. The *questionnaire* follows the reference of the RPA official codes and the possibility of specific post-graduate training.

The answers of 168 professionals indicate a gap in seismic knowledge between architects and engineers. They also indicate dissatisfaction with the availability of training, particularly by architects. The survey also sheds light on a difference in the interpretation of the fundamental principles of seismic design.

## Presentation of the sample

Despite the wide distribution, we received only 168 completed *questionnaires*:

- 107 answers from architects
- 61 answers from engineers.

These two functions form a common statistical sample in order to maintain a statistical interpretation.

### Professionals’ place of higher education

Table 1 shows the location of universities and schools where the respondents studied architecture or civil engineering.

**TABLE 1:** High education place of study of architects and engineers.

Place of study	Degree		
	Architects	Engineers	Total
Epau Alger	18	0	18
Blida	14	5	19
Oran	10	0	10
Constantine	0	3	3
Laghouat	24	11	35
Mostaganem	9	3	12
Tizi Ouzou	8	2	10
Setif	9	0	9
Biskra	7	0	7
Tlemcen	4	1	5
Batna	1	0	1
Bejaia	3	0	3
Inforba	0	8	8
Ecole Polytechnique Alger	0	6	6
Usthb Alger	0	7	7
Tiaret	0	15	15

**Total**	**107**	**61**	**168**

It can be seen that:

- For the architects:
  ■ All the universities and schools qualified to train architects are represented, except those without a first-year graduation.
  ■ Laghouat University holds the highest number of participants, with a rate of 22%, which does not reflect a distinctive adherence to our survey but is simply explained by ‘door-to-door’ and the personal awareness effort related to the proximity of Laghouat BETs to our own workplace.
- For the engineers
  ■ All of the regions are represented, despite the low participation of BET engineers in the survey.
  ■ Participation by study location ranges from 2% to 18%.
- The over-representation of Laghouat University (18% of responses) is explained by the same rationale as for architects.

### Profession region of practice

**FIGURE 1:**
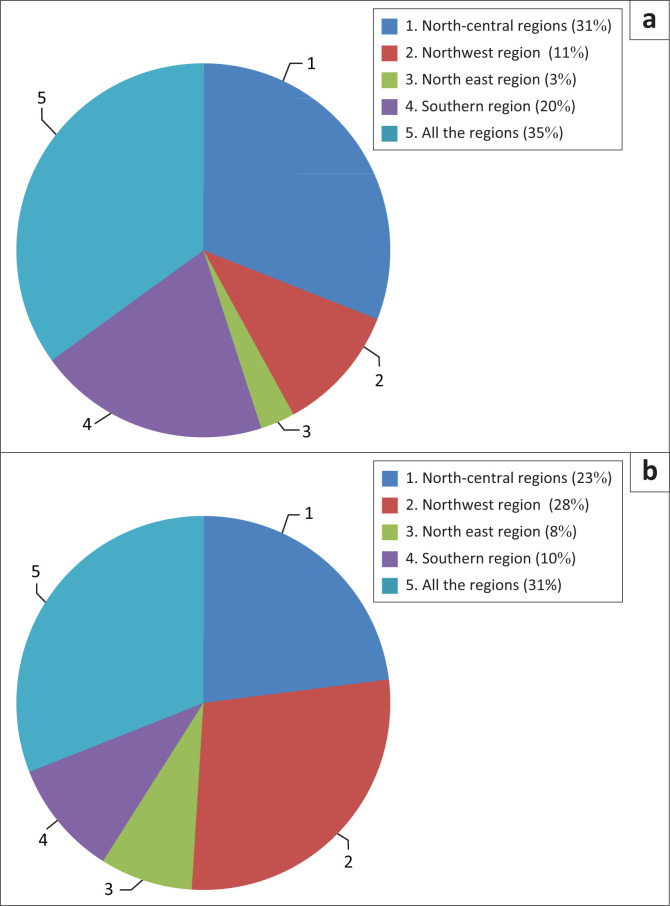
Profession region practice. (a) Architects, (b) Engineers.

To assess the perception of seismic engineering at the BET level, we asked architects and engineers about their areas of intervention, as shown in Figure 1.

This treatment shows that all regions are represented in our sample but in varying proportions. However, this variation does not influence the study. Eighty percent of BETs work in the Algerian northern regions, where seismic zoning indicates moderate and high seismicity. This observation supports our objective, since the consideration of seismic risk in construction in these regions should be more rigorous.

### Architects’ and engineers’ professional experience

This question aims to measure the influence of the experience capitalised at the BET level on the consideration of seismic risk, as shown in Table 2.

**TABLE 2:** Number of years of professional experience.

Number of years of experience	Degree		
	Architects	Engineers	Total
1 to 5 years	32	1	33
6 to10 years	36	12	48
11 to15 years	26	23	49
16 to 20 years	12	16	28
More than 20 years	1	9	10

**Total**	**107**	**61**	**168**

From the above, we deduce that the experience of the respondents is sufficient for our study, since 70% of architects and 90% of engineers have between 6 and 20 years of practice.

### Analysis of responses related to seismic protection

The following analysis summarises the survey responses and information from interviews with construction professionals (project owners, contractors and technical inspectors).

### Consideration of the seismic hazard

**FIGURE 2:**
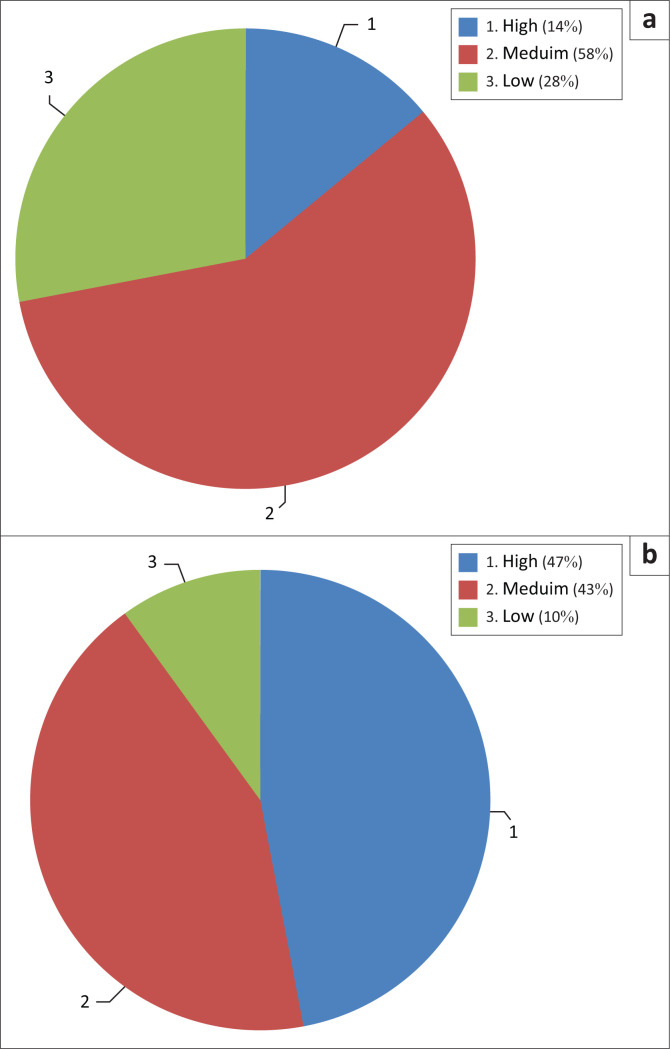
Consideration of seismic hazard by professionals of Bureau d’Etudes Techniques (BET), according to architects and engineers. (a) Architects, (b) Engineers.

When asked about the consideration of seismic risk in their professional activity, architects and engineers report the impressions as laid out in Figure 2.

- From this distribution, we can see:
  ■ **For architects:** The majority (58%) consider that seismic hazard is integrated in a ‘medium’ way, a quarter (28%) consider seismic concern ‘low’, and only a few (14%) declare a ‘high’ concern in their professional field (Figure 2a).
  ■ **For engineers:** Forty-three percent consider the concern ‘medium’, 47% ‘high’ and 10% consider that the concern for this hazard is ‘low’ (Figure 2b).

The clear contrast between the declarations of architects and engineers is explained by the difference in basic training and the difference of tasks for each profession. Twenty percent of architects and engineers consider that seismic hazard is not very integrated in the exercise of their activity. We can deduce this in the absence of a general consideration about seismic hazard, which varies according to the BETs.

### Appliance of anti-seismic engineering in the profession

The question, ‘How would you describe the consideration of seismic engineering in the practice of your profession?’, is related to the level of consideration of seismic engineering in the design of structures.

**FIGURE 3:**
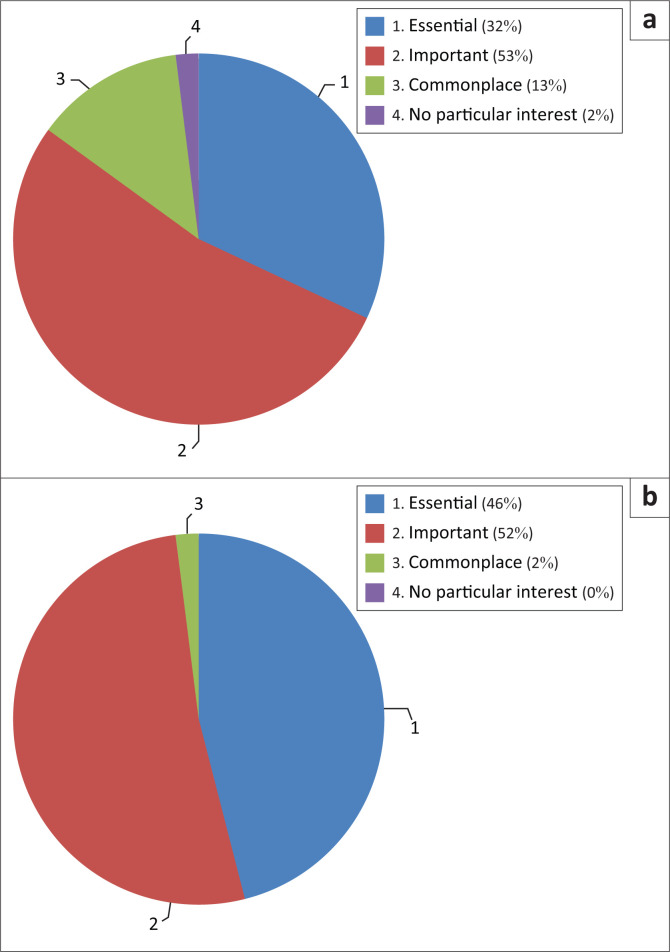
Consideration of seismic engineering in the design. (a) Architects, (b) Engineers.

- The answer to this question show that:
  ■ **For architects:** Eighty-five percent of architects describe seismic design as ‘essential’ or ‘important’, but 13% consider this integration to be ‘commonplace’ and only 2% consider it to be ‘of no particular interest’ (Figure 3a).
  ■ **For engineers:** The results are equivalent to those of the architects, since 98% describe this integration as ‘important’ or ‘essential’. However, the answers are not equivalent for those who consider this integration to be ‘banal’ or even ‘irrelevant’ (Figure 3b).

Despite this general trend towards seismic design, it remains that 16% of architects describe seismic design as ‘banal’ or ‘uninteresting’.

### Training in seismic engineering

**FIGURE 4:**
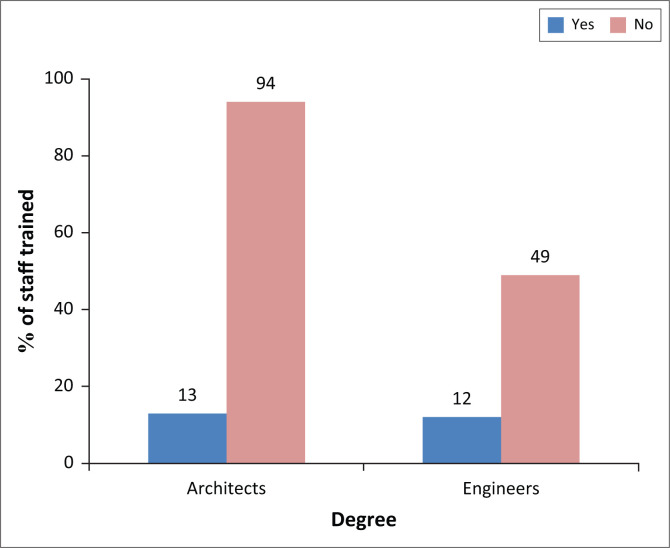
Seismic engineering training.

The question about the training of architects and engineers on seismic hazard and seismic engineering gives the following result: It can be seen from Figure 4 that the majority of managers in the BETs, that is, more than 87% of architects and more than 80% of engineers, have not received training in earthquake engineering to improve their knowledge and to ensure proper application of earthquake codes. Indeed, the overall rate for the two professions, which amounts to more than 85%, corresponds to those without post-graduate seismic training.

Based on interviews with other professionals in the sector, we believe that this rate accurately represents these two trades and accurately reflects the situation of seismic training, particularly amongst architects. This alarming result requires further research into the real reasons for this situation, given the omnipresent seismic hazard in the northern Algerian regions.

Another question concerns the reasons for the lack of training in BETs. Table 3 presents the results.

**TABLE 3:** Reasons for the lack of seismic training in BETs (Bureau d’Etudes Techniques).

Reasons for not following seismic training	Degree		
	Architects	Engineers	Total
Lack of time	13	4	17
No interest	2	0	2
Lack of training opportunities	79	45	124

**Total**	**94**	**49**	**143**

It should be noted that almost all of the architects and engineers interviewed without seismic training (almost 87%) attribute it to the absence of training opportunities in this field.

For those trained, one question concerns the type and level of satisfaction of the training. The results are presented in Tables 4 and 5.

**TABLE 4:** Type of training.

Type of training	Degree		
	Architects	Engineers	Total
Short-term Training	5	5	10
Seminar	8	7	15

**Total**	**13**	**12**	**25**

**TABLE 5:** Level of satisfaction with training.

Level of satisfaction	Degree		
	Architects	Engineers	Total
Satisfied	11	9	20
No satisfied	2	3	5

**Total**	**13**	**12**	**25**

From these two tables, the following observations can be made:

- The absence in our sample of people who have undergone long-term training in this field, which sheds light on the current state of professional seismic training, particularly amongst architects.
- The rare training courses are seminars and study days.
- Engineers are more used to seminars and study days than architects (20% vs. 12%).
- Amongst those who have attended these trainings, 80% say they are satisfied.

### Level of knowledge of the seismic code goals

The following questions concern the state of knowledge in seismic engineering. So that these questions do not appear as an academic examination which could lead to a refusal to participate in the survey, we deliberately avoided purely technical questions linked to the behaviour of the constructions or the causes of the damage.

Thus, the question on the knowledge of regulation goals includes four items whose answers are assessed in the light of the official doctrine of the Algerian anti-seismic code (Ministry of Housing and Town Planning 2003). As with all seismic codes, RPA sets the survival of inhabitants or users as the main objective of building safety, which induces accurate or inaccurate answers.

**Item 1:** Is the objective of seismic rules to avoid serious disorders?

The answers to this question, which are shown in Table 6, show that 10% of respondents think that ‘avoiding serious disorders’ is not the main objective of seismic rules. The sorting of the answers shows that this rate is 11% amongst engineers, which is equivalent to that of architects (10%). However, this rate should be as low as possible, since engineers use seismic codes more than architects and should therefore know their objectives better. This unexpected result can be explained by a nuance of personal interpretation: the ‘false’ answer of this group of engineers is correct if it has been specified that the magnitude of the quake is very high.

**TABLE 6:** Objective 1 of seismic rules (avoid serious disorders).

Objective1: Avoid serious disorders	Degree		
	Architects	Engineers	Total
True	96	54	150
False	11	7	18

**Total**	**107**	**61**	**168**

**Item 2:** Is the objective of seismic codes to avoid any disorder?

In the light of the answers to this second question, as shown in Table 7, it can be said that despite 90% correct answers to the first question (item 1), those related to the second question (item 2) are mixed, as the correct answers represent only 52% for architects and 68% for engineers. The inaccurate responses, therefore, represent an average of 43% for both profiles, which we believe is particularly high.

**TABLE 7:** Objective 2 of seismic rules (avoid disorder).

Objective2: Avoid any disorders	Degree		
	Architects	Engineers	Total
True	51	20	71
False	56	41	97

**Total**	**107**	**61**	**168**

**Item 3:** Is the objective of seismic codes to guarantee that no collapse occurs?

The question seems to be well understood by respondents, particularly engineers, since 74% of architects’ responses are accurate and 95% of engineers’ responses are accurate (Table 8). However, those who did not respond correctly represent a significant part, particularly for architects, as this is an essential principle of the philosophy of seismic codes.

**TABLE 8:** Objective 3 of seismic rules (guarantee that the building do not collapse).

Objective3: Guarantee the non-collapse of buildings	Degree		
	Architects	Engineers	Total
True	79	58	137
False	28	3	31

**Total**	**107**	**61**	**168**

**Item 4:** Is the objective of seismic rules to guarantee the safety of all the people?

As shown in Table 9, the correct answer to this question represents 83% of architects and 90% of engineers. Despite its small proportion, the wrong answer can support the hypothesis about the basic level of knowledge about objectives of seismic regulation.

**TABLE 9:** Objective 4 of seismic rules (guarantee the safeguarding of all people).

Objective4: Guarantee the safeguarding of all people	Degree		
	Architects	Engineers	Total
True	88	55	143
False	19	6	25

**Total**	**107**	**61**	**168**

This analysis encourages the examination of the correct answers to the four items on the objectives of seismic codes amongst the 25 professionals who received seismic training.

This correlation represented in Table 10 shows that engineers and architects who have received training in seismic engineering and who work in BETs, answer almost (more than 90%) correctly to this basic question. It clearly indicates the effect of the training and the understanding of the philosophy of seismic codes.

**TABLE 10:** Seismic training and correct answers for code objectives.

Reminder of the objective	Number of architects and engineers trained in seismic design and the correct answers				Total		Rate (%)
	Engineers	Number of correct answers	Architects	Number of correct answers	Architects and engineers	Number of correct answers	
Avoid serious disorders	12	11	13	12	25	23	92
Avoid any disorders	-	12	-	12	-	24	96
Guarantee the non collapse of buildings	-	12	-	12	-	24	96
Guarantee the safeguarding of all people	-	11	-	11	-	22	88

Amongst BET engineers and architects who received seismic training, more than 90% answer accurately about regulatory objectives. This observation clearly demonstrates the positive effect of training.

### Construction and seismic code

Two questions concern the resistance of buildings to quakes and the occurrence of structural damage for buildings calculated according to seismic codes, which had been implemented correctly. The first item (Effect 1, Table 11) examines if the construction calculated according to seismic codes is supposed to resist without collapsing in all destructive earthquakes. The second item (Effect 2, Table 12) is to see if this same structure is not expected to suffer structural damage. The exact answers to these questions are obviously ‘wrong’ for both questions, but the answers collected are tabulated below.

**TABLE 11:** Effect 1 (resist without collapse).

Effect 1: Supposed to resist without collapsing in all destructive earthquakes	Degree		
	Architects	Engineers	Total
True	85	34	119
False	22	27	49

**Total**	**107**	**61**	**168**

**TABLE 12:** Effect 2 (suffers damage).

Effect 2: Is not expected to suffer structural damage	Degree		
	Architects	Engineers	Total
True	81	35	116
False	26	26	52

**Total**	**107**	**61**	**168**

For these two questions, we only recorded an average of 30% of correct answers for both profiles, with a clear difference in favour of engineers. This result seems paradoxical considering the rates of correct answers to the previous questions, which leaves us at this stage of analysis to affirm the inadequacy of seismic knowledge, particularly for architects.

Summary Table 13 below, focusing on professionals trained in seismic codes, highlights the positive effect of training on these results.

**TABLE 13:** Seismic training and correct answers for the resistance of buildings to earthquakes.

Reminder of the question	Number of architects and engineers trained in seismic design and the correct answers				Total		Rate (%)
	Engineers	Number of correct answers	Architects	Number of correct answers	Architects and engineers	Number of correct answers	
**Effect 1:** Supposed to resist without collapsing in all destructive earthquakes	12	11	13	11	25	22	88
**Effect 2:** Is not expected to suffer structural damage	-	11	-	10	-	21	84

Almost all seismic training beneficiaries give correct answers to these two questions, although incorrect answers still represent 70% of the total sample.

### Creativity in architectural expression and seismic codes

This question identifies the relationship between seismic design and architectural creativity.

The responses in Table 14 show that 66% of architects and 52% of engineers surveyed believe that seismic codes and design inhibit the artistic expression of forms, structural and material choices.

**TABLE 14:** Limitation of creativity in architectural expression.

Limiting Creativity in Architectural Expression	Degree		
	Architects	Engineers	Total
True	71	32	103
False	36	29	65

**Total**	**107**	**61**	**168**

Indeed, to the open-ended question asking to explain this conviction, the majority mention:

- Limitation in shapes (plan and height configuration)
- Limitation on the choice of structures
- Limitation on building materials
- The presence of the shear walls
- Oversizing of pillars and beams.

These opinions express a ‘preconceived notion’ among the majority of responding professionals. It is important to correct it through initial or additional seismic training of architects and engineers.

### Seismic construction and additional costs

**FIGURE 5:**
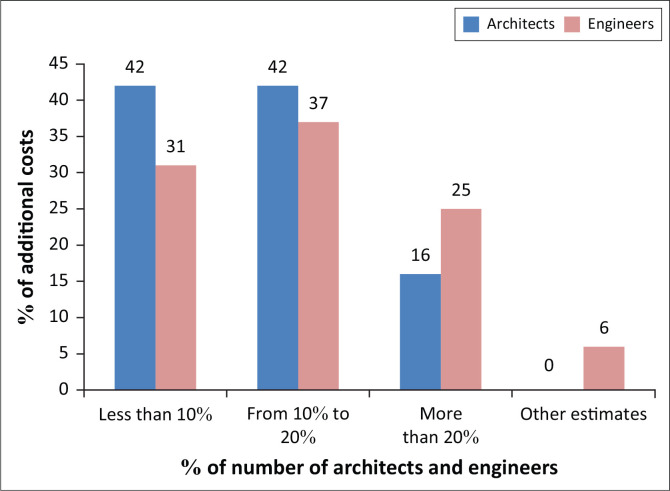
Estimation of the additional costs of seismic construction.

Two questions concern the additional costs that seismic construction can induce. The first is whether new seismic construction actually induces additional costs. The second is reserved for those who respond with a statement, to give an estimate in percentage terms of the project cost. The responses are provided in Table 15 and Figure 5.

**TABLE 15:** Additional costs of seismic construction.

Additional costs of seismic construction	Degree		
	Architects	Engineers	Total
True	76	32	108
False	31	29	60

**Total**	**107**	**61**	**168**

Seventy-one percent of architects and 52% of engineers consider that seismic construction necessarily entails additional costs.

The additional costs are overestimated for seismic construction. This supports the hypothesis that the majority of architects and engineers in the BETs do not know the basics of earthquake-resistant construction.

### Seismic damage and compliance with Algerian paraseismic rules – rules

We try to determine the opinion of BET professionals regarding seismic damage to buildings in general, that is, whether or not it is the result of incorrect application of RPAs or partial or total non-compliance with requirements. The answers are presented in Table 16.

**TABLE 16:** Seismic damage and Algerian paraseismic rules – compliance.

The seismic damage is generally result of non-compliance with RPA	Degree		
	Architects	Engineers	Total
True	84	38	122
False	23	23	46

**Total**	**107**	**61**	**168**

Almost 80% of architects and more than 62% of engineers say that seismic damage results from non-compliance with RPA rules; however, post-seismic surveys reveal causes other than direct non-compliance with these rules:

- Architectural design
- Incorrect adequacy with geology and relief
- The impact of neighbouring buildings.

The open-ended question about the other causes offers some disparate results:

- Fifty percent abstained from answering
- Twenty-three percent blame faulty execution and unskilled workers
- Twenty-seven percent blame the weakness of monitoring and control on the site.

### Qualification of the implementing company for the application of Algerian paraseismic rules – code

This question (Do you consider that the companies are well supervised for the application of the RPA codes?) aims to obtain the opinion of architects and engineers from the BETs who supervise the worksites and the qualification of technical staff for the application of RPAs. The result is presented in Table 17.

**TABLE 17:** Guidance of companies for the application of Algerian paraseismic rules.

Supervision of companies for the application of RPA	Degree		
	Architects	Engineers	Total
True	1	0	1
False	106	61	167

**Total**	**107**	**61**	**168**

RPA, Algerian paraseismic rules.

Almost all architects and engineers say that building companies are not well supervised for the application of RPA codes.

This unanimous concern for the lack of seismic supervision in construction companies accentuates the lack of seismic training for designers (both architects and engineers).

The open-ended question indicates that these companies must, in order to apply seismic codes, have:

- Qualified staff
- The competence of technical managers
- The continuous training of technical staff
- The supervision of the worksites by engineers, etc.

We observed a dissonance in the responses of the majority of the sample on the seismic training component, since the same respondents acknowledge this inadequacy in their own careers.

The responses to the question on the recommendations of the respondents express an expectation of:

- Qualification and training of company workers
- Rigour in the control asserted by the authorised bodies.

These suggestions seem insufficient, because these professionals do not seem interested in architectural anti-seismic design and they do not propose any action to improve their knowledge of seismic engineering.

### Architects and seismic design

When asked if architects feel themselves well trained in seismic design, 90% answer ‘no’. This result alerts us to the recognition by the design architects themselves of their shortcomings in seismic design.

In this regard, it is demonstrated that architectural design is as important as the application of seismic codes (Association Française du Génie Parasismique 2004). The behaviour of a structure under a shake is determined practically upstream of the codes by the geometry of the sketch (i.e., the distribution of masses and rigid elements and the type of structure). The architect must therefore have solid seismic knowledge. This baggage indicates, before the calculation, optimal conditions for earthquake resistance. He ‘owes’ to his client an irreproachable tailor-made service (Zacek & Balandier 2003).

In addition, rational building design reduces the cost of seismic protection.

### Suggestions and recommendations of the respondents

At the end of our investigation, an open-ended question allows expression of recommendations about the application of seismic engineering. Seventy percent of the responses indicated the priorities below:

- More control on the worksites
- More qualifications required from companies
- Check the quality of materials.

## Conclusion

This study points out some deficiencies, inadequacies and even inconsistencies in the seismic culture of the architects and engineers working in the design offices in Algeria. It also shows the need for seismic training for professionals to design and control buildings according to the Algerian seismic code (Ministry of Housing and Town Planning 2003).

Indeed, the current university education of architects only addresses the specific rules of construction in seismic zones in an allusive way. Architects generally leave it to engineers to reconcile their works with the calculation at the step of design. This represents a weak seismic approach that can have dramatic consequences and induce expensive additional costs.

Post-graduate training in seismic design is the best way for architects to be informed continuously. This training gives these designers specific expertise and optimises the safety of the structure.

Finally, the specifications of the owner should include seismic design from the outset of the project.
